# Molecular evolution of acetohydroxyacid synthase in bacteria

**DOI:** 10.1002/mbo3.524

**Published:** 2017-08-06

**Authors:** Yadi Liu, Yanyan Li, Xiaoyuan Wang

**Affiliations:** ^1^ State Key Laboratory of Food Science and Technology Jiangnan University Wuxi China; ^2^ School of Biotechnology Jiangnan University Wuxi China; ^3^ Synergetic Innovation Center of Food Safety and Nutrition Jiangnan University Wuxi China

**Keywords:** acetohydroxyacid synthase, AHAS, BCAA biosynthetic pathway, molecular evolution, phylogenetic trees

## Abstract

Acetohydroxyacid synthase (AHAS) is the key enzyme in the biosynthetic pathways of branched chain amino acids in bacteria. Since it does not exist in animal and plant cells, AHAS is an attractive target for developing antimicrobials and herbicides. In some bacteria, there is a single copy of AHAS, while in others there are multiple copies. Therefore, it is necessary to investigate the origin and evolutionary pathway of various AHASs in bacteria. In this study, all the available protein sequences of AHAS in bacteria were investigated, and an evolutionary model of AHAS in bacteria is proposed, according to gene structure, organization and phylogeny. Multiple copies of AHAS in some bacteria might be evolved from the single copy of AHAS, the ancestor. Gene duplication, domain deletion and horizontal gene transfer might occur during the evolution of this enzyme. The results show the biological significance of AHAS, help to understand the functions of various AHASs in bacteria, and would be useful for developing industrial production strains of branched chain amino acids or novel antimicrobials.

## 1 INTRODUCTION

**Figure 1:**
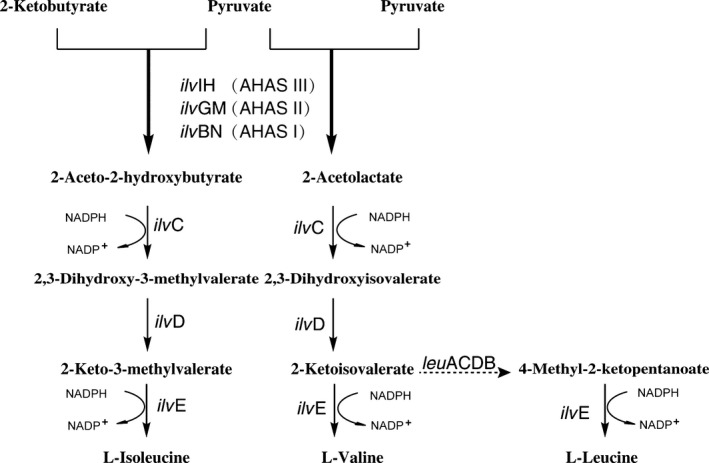
Branched chain amino acid pathway in *Escherichia coli*

Acetohydroxyacid synthase (AHAS) is capable of catalyzing the synthesis of either acetolactate from pyruvate or 2‐aceto‐2‐hydroxybutyrate from pyruvate and 2‐ketobutyrate. It is the key enzyme of the metabolic pathways leading to the synthesis of branched chain amino acids (BCAA) (Figure 1). The affinity of AHAS to 2‐ketobutyrate or pyruvate plays a key role in determining the relative fluxes to different end products (Epelbaum et al., 1998; Yin et al., 2012; Chen, Li, Hu, Dong, & Wang, 2015). Many gram‐positive bacteria express a single AHAS, But some gram‐negative bacteria such as *Escherichia coli*,* Salmonella typhimurium* encode three isozymes of AHAS (AHAS I, II, and III) that differ in their biochemical properties, including sensitivity to inhibition by valine, specificity toward substrates, affinity to combine cofactors and different regulation ways of expression (Barak & Chipman, 2012). Each AHAS presumably has a slightly different metabolic role.

In bacteria, AHAS is composed of large subunits (AHAS‐L) and small subunit (AHAS‐S) in equimolar amounts, which is coded by a pair of linked genes. Sequence and structure analyses have revealed that each AHAS‐L (~60 kDa) has similar size, while the size of AHAS‐S ranges from 9 to 17 kDa. ACT (named after the first letters of three proteins: aspartate kinase AK, chorismate mutase CM, and prephenate dehydrogenase TyrA) is a structural motif in proteins that function in the control of metabolism, solute transport, and signal transduction (Grant, 2006; Chipman & Shaanan, 2006). In *E. coli*, the small subunit of AHAS III encoded by the gene *ilv*H contains two ACT‐like subdomains, while the small subunits of AHAS I and AHAS II contain only one ACT‐like subdomain (Kaplun et al., 2006; Vinogradov et al., 2006; Barak & Chipman, 2012). In addition, isozymes of AHAS seems to show different inhibition by L‐valine. *Escherichia coli* AHAS II encoded by *ilv*GM is insensitive to the feedback inhibition by L‐valine even under saturating concentrations, while AHAS I encoded by *ilv*BN can be completely inhibited by L‐valine. These particular structure pattern and various inhibition of L‐valine have raised the question of how and why different AHASs emerged in the course of bacterial evolution. Because its sensitivity to the terminal BCAA products, understanding AHAS is important to improve BCAA production in industrial production strains. From an evolution point of view, enzymes like AHAS which could simultaneously participate in two pathways are particularly significant. Threonine dehydratase, another key enzyme in the biosynthetic pathway of L‐isoleucine, has been reported to occur horizontal gene transfer, gene fusion, duplication and deletion during the evolution (Yu, Li, & Wang, 2013). The *leu* genes in the BCAA biosynthetic pathway are paralogous to *lys* and *arg* genes, and their interrelationships might due to a cascade of duplication of ancestral genes which could catalyze different kinds of substrates (Fondi, Brilli, Emiliani, Paffetti, & Fani, 2007a). But the evolutionary mode of AHAS in bacteria has not been reported.

AHAS belongs to the thiamin diphosphate (ThDP)‐dependent enzyme family (Chang & Cronan, 1988). An evolutionary pathway of ThDP‐dependent enzymes has been depicted (Duggleby, 2006; Costelloe, Ward, & Dalby, 2008). Domain recruitment, domain linkage and structure rearrangement of catalytic domains are proposed to explain the sequence and structure diversity of the whole ThDP‐dependent family (Vogel & Pleiss, 2014). The evolutionary mode of ThDP‐dependent family, and the available sequence and structure of AHAS in several bacteria would be helpful for us to understand the evolution of AHAS (Pang, Duggleby, Schowen, & Guddat, 2004; Tittmann, Vyazmensky, Hübner, Barak, & Chipman, 2005; Vinogradov et al., 2006; Baig, Moon, Kim, Koo, & Yoon, 2014; Sommer et al., 2015). In this study, we analyzed the amino acid sequences of the available AHAS in bacteria, constructed phylogenetic trees, and proposed an evolutionary pathway leading to the genes coding AHAS in the present bacteria. In addition, the similarity and important sites of different copies of AHASs were also analyzed to clarify the relationship of AHASs.

## 2 MATERIALS AND METHODS

### 2.1 Distribution of bacterial strains containing AHAS and construction of phylogenetic trees

The distribution of species containing AHAS in nature were obtained from the UniProtKB database (http://www.uniprot.org/uniprot/?query=2.2.1.6&sort=score#orgViewBy) (Consortium, 2014) by searching “EC 2.2.1.6.” In this database, from the taxonomy, AHAS were found in 21,576 cellular organisms, those include 19,708 species in bacteria, 1,204 species in Eukaryotes and 664 in Archaea. Because of the distribution in bacterial species are mainly in Proteobacteria (11,022), Firmicutes (4,540) and Actinobacteria (2,243), 70 genomes were selected from those three phyla, and in most cases only one strain for each species was taken into account. The amino acid sequences of the *E. coli* AHAS I, II, III and *Corynebacterium glutamicum* AHAS were used as a query to probe the 70 genomes with the BLASTP option of BLAST program in order to retrieve the most similar sequences (Altschul et al., 1997). The representative species and the AHAS they contain are listed in TableS1. These AHAS were divided into groups by isozymes in enterobacteria and different phylogenetic branch of AHAS in species of firmicute and actinobacteria. The 16S rDNA of 70 selected species were download from Ribosomal Database Project (Cole et al., 2013). Protein sequences of AHAS and 16S rDNA sequences of 70 genomes were, respectively aligned by ClustralX2.1 (Larkin et al., 2007). Then those aligned files were performed using Mega 5 software using the neighbor‐joining methods, meanwhile 1,000 bootstrap replicates, complete deletion, and poison correction were selected during the performance (Kumar, Nei, Dudley, & Tamura, 2008).

### 2.2 Sequence and structure alignments of different AHAS

There are 17,205 AHAS sequences in the protein database of UniprotKB database. The genes coding AHAS‐L and AHAS‐S are both included in the retrieved results. Thus, the sequence length more than 500 amino acid is another important filter. From KEGG database, through searching “EC 2.2.1.6”, gene and protein information of AHAS in different organisms were shown clearly. Due to errors in annotation and confusing nomenclature issues that have been perpetuated in the databases, some organisms contain only one AHAS, while some organisms contain more than eight AHAS which have low sequence similarity with each other. So those AHAS which are completely familiar with the branched chain amino acids pathway and are familiar in their scholarly literature were selected in this study. After downloading these AHAS sequences from 70 genomes, the alignments of these AHAS were performed using ClustralX 2.1 (Larkin et al., 2007), and the logos were generated using Weblogo 3 web service by input AHAS alignment files (http://weblogo.threeplusone.com/create.cgi) (Crooks, Hon, Chandonia, & Brenner, 2004). The crystal structure of AHAS I‐S (2LVW) and AHAS III‐A‐S (2F1F) from *E. coli* were obtained from PDB database (Berman et al., 2002). These structures were used to build the comparison model by PyMol.

### 2.3 Sequence retrieval of other enzymes among branch chain amino acids biosynthesis pathway

Amino acid sequences of enzymes among branch chain amino acids biosynthesis pathway were retrieved from GenBank database. BLAST probing of each enzymes from database was performed with the BLASTP option of this program. Meanwhile, from KEGG SSDB Gene cluster, the KEGG ID of genes next to AHAS in each genome could be shown directly.

## 3 RESULTS

### 3.1 Four types of AHAS‐L exist in bacteria according to the distribution analysis

**Figure 2:**
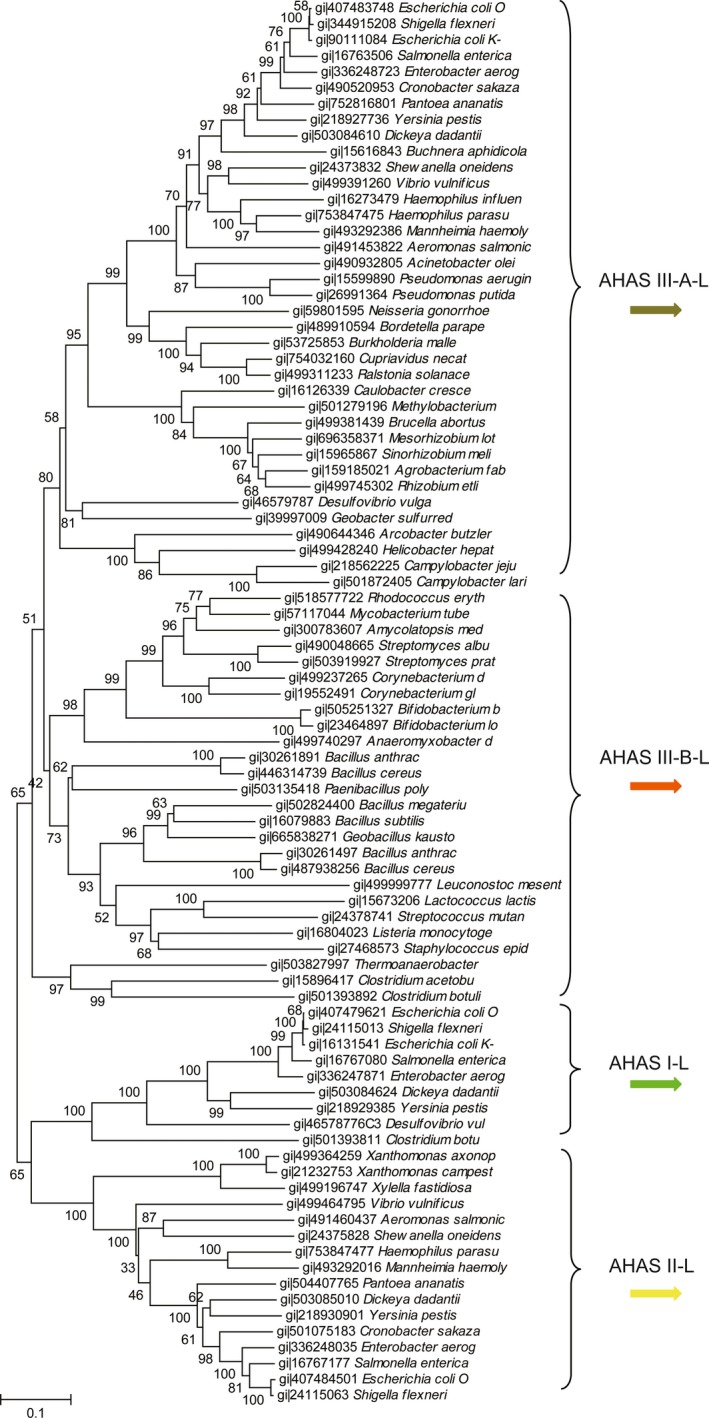
Phylogenetic tree constructed on AHAS‐L sequences from 70 representative species. Different genes encoding AHAS‐L are represented by arrows with different colors. AHAS, acetohydroxyacid synthase

Based on UniProtKB database, AHAS are widely distributed in 21,576 species, i.e., 91% in bacteria, 6% in eukaryotes and 3% in archaea. In bacteria, they are mainly distributed in Proteobacteria (60%), Firmicute (21%), and Actinobacteria (10%). Seventy organisms selected from these three phyla as representative species (45 in Proteobacteria, 16 in Firmicutes and 9 in Actinobacteria) were used for the phylogenetic analysis. A phylogenetic tree was constructed using the large subunit protein sequences of AHAS from 70 bacterial species (Figure 2). Overall, four major clusters were observed in the tree, and they were denominated as AHAS III‐A‐L, AHAS III‐B‐L, AHAS I‐L, and AHAS II‐L. Since AHAS sequences used for constructing the tree were selected from a wide range of species, some bootstrap values on the tree are lower than 50. Among the four clusters, AHAS III‐A‐L is close to AHAS III‐B‐L, AHAS II‐L is close to AHAS I‐L. Therefore, AHAS III‐A‐L and III‐B‐L might share the common ancestor, while AHAS I‐L and II‐L might be derived from a common ancestor.

Among AHAS III‐A‐L cluster in Figure 2, there are protein sequences of single AHAS from α‐, β‐, δ‐, and ε‐ proteobacteria species, and protein sequences of one of three isozymes from γ‐ proteobacteria. Because the sequence of single AHAS formed a unique cluster with AHAS among three isozymes, horizontal gene transfer might not strongly influence the evolution of AHAS III‐A in proteobacteria, and AHAS III‐A might be appeared before the divergence of new species. AHAS III‐B‐L cluster was found in species of firmicutes and actinobacteria. In Figure 2, AHAS III‐B‐L cluster formed a unique cluster aside from AHAS III‐A‐L. The phylogenetic tree constructed using sequences of AHAS‐S (Figure S1) shows the similar pattern to Figure 2, but detailed difference can be observed. For example, AHAS III‐B‐S protein sequences of actinobacteria species clustered closer to proteobacterial sequences AHAS III‐A‐S, suggesting that the evolution of AHAS III‐A and III‐B share the common ancestor but later divergence occurred to satisfy the demand of different species.

The sequences of other AHAS copies in γ‐proteobacterial were separately clustered in AHAS I‐L and II‐L cluster which contain the reported AHAS I and AHAS II in *E. coli* (Wek, Hauser, & Hatfield, 1985; Lawther et al., 1987), respectively. The AHAS‐L in *Xanthomonadaceae* represented an exception to genes in AHAS III‐A‐L. It had a single AHAS, but it was clustered in AHAS II‐L. The exceptions in *Xanthomonadaceae* could also be observed in the evolution model of aspartokinases and threonine dehydratases (Fondi, Brilli, & Fani, 2007b; Yu et al., 2013). This exception may cause by horizontal gene transfer or delete genes in the genome. The genes coding AHAS were located in the operon, it was deduced that AHAS could be generated by horizontal gene transfer from other species with the operon. The biological significance of the cluster operon might rely in the expansion and the refinement of ancestral metabolic routes. Ancestral enzymes which possessed broad substrate specificity try to form well defined routes to produce a predefined output (Fani & Fondi, 2009).

### 3.2 Distribution of genes encoding AHAS in bacteria

**Figure 3:**
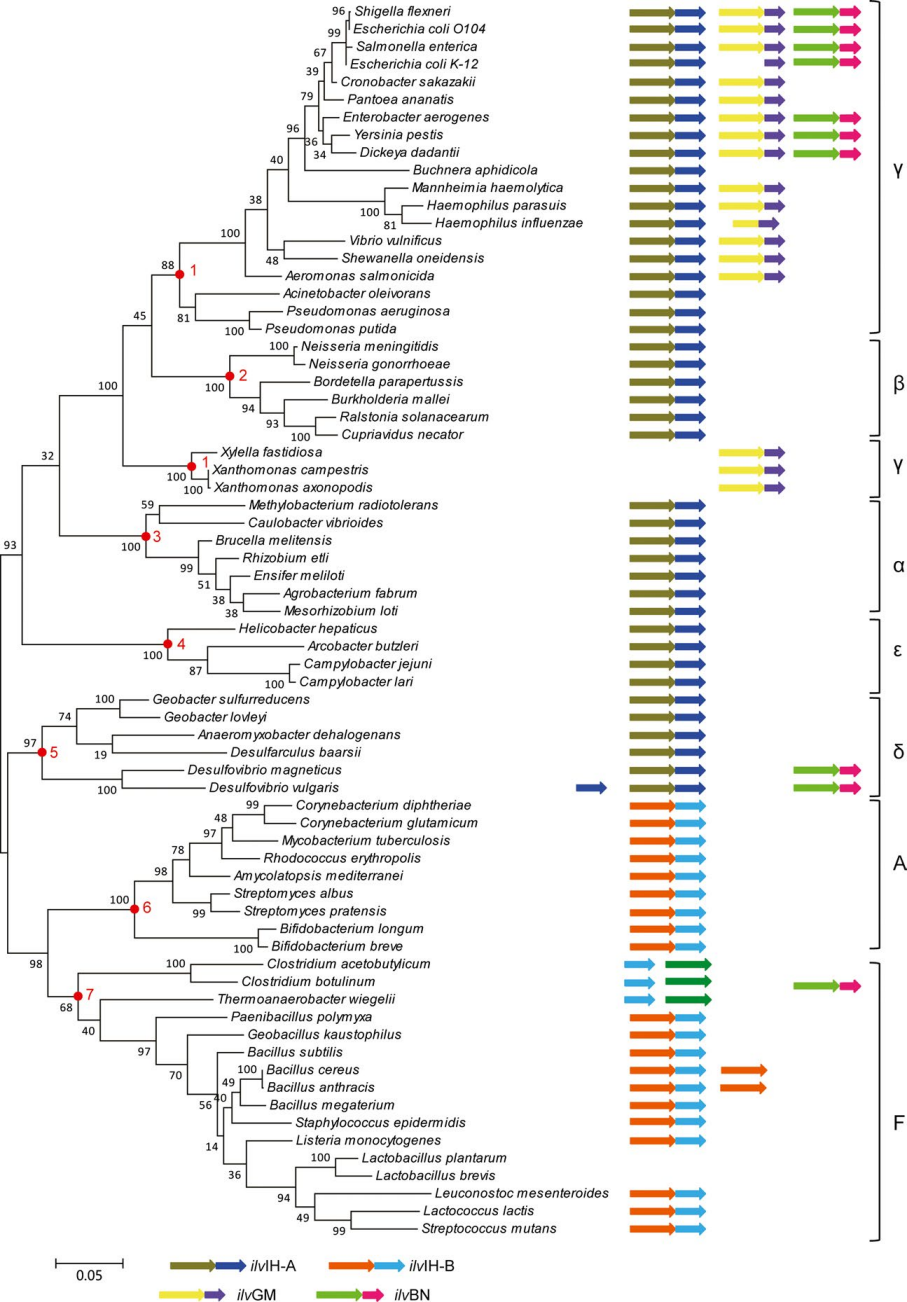
Distribution of acetohydroxyacid synthase (AHAS) among bacteria. The 16S rDNA tree is shown at the left. The scale bar indicates 0.05 change per nucleotide. Circled node positions from 1 to 7 indicate Proteobacteria (the classes of Gamma‐, Beta‐, Aplha‐, Eplison‐, and Delta‐), Actinobacteria, and Firmicutes, respectively. The arrows at the right represent the genes coding AHAS that could exist in the bacterium. Note that only the presence or absence of genes, not gene order is indicated

Data obtained in Figure 2 were schematically reported in Figure 3, where a phylogenetic tree constructed using the 16s rDNA sequences of the 70 species was shown together with the number and structure of all the retrieved AHAS coding genes. The internal node positions in this Figure 3 are congruent with the protein phylogenetic tree in Figure 2. In all α‐, β‐, δ‐, and ε‐proteobacterial genomes, a single copy of AHAS was detected, except for the genus of *Desulfovibrio* in δ‐proteobacterial. It had two copies of AHAS, one was common to the single copy in other genomes, the other was thought to be similar with the isozyme AHAS I in enterobacteria by pblast, and it was clustered in AHAS I in Figure 2. Meanwhile, in Figure 3, there was a single copy of AHAS in firmicutes and actinobacteria like AHAS III‐A in α‐, β‐, δ‐, and ε‐ proteobacteria species. Based on these data, AHAS III‐A and AHAS III‐B might have the common ancestor. Then the subsequent evolution of AHAS III was congruous with the evolutionary divergence of species.

AHAS I‐L and II‐L cluster protein sequences were found mainly in γ‐proteobacteria, where the scenario was more complex and intriguing. One, two, or even three copies of AHAS could be observed in the γ‐proteobacteria. The absence of multi‐copies AHAS genes was occurred in *Pseudomonas* and *Acinetobacter*. In spite of their taxonomical position within γ‐proteobacteria, they exhibited the same structural and organization pattern of bacteria belonging to α‐, β‐, δ‐, and ε‐subdivisions. This was not an isolated example, this situation had been recorded for other enzymes, such as aspartokinases and histidinol‐phosphate phosphatase (Brilli & Fani, 2004; Fondi et al., 2007b). The reason of such distribution was still unclear. Moreover, there was an apparent increasing complexity concerning these genes that were parallel to the evolutionary branching of γ‐proteobacteria, with enterobacteria showing the highest number of copies of AHAS. The exception of *E. coli* K‐12 which showed no AHAS II‐L was caused by frame shift mutation inducing errors in the normal translation order (Lawther et al., 1987; Park & Lee, 2010).

### 3.3 Conservation of amino acid sequences among different types of AHAS in bacteria

**Figure 4:**
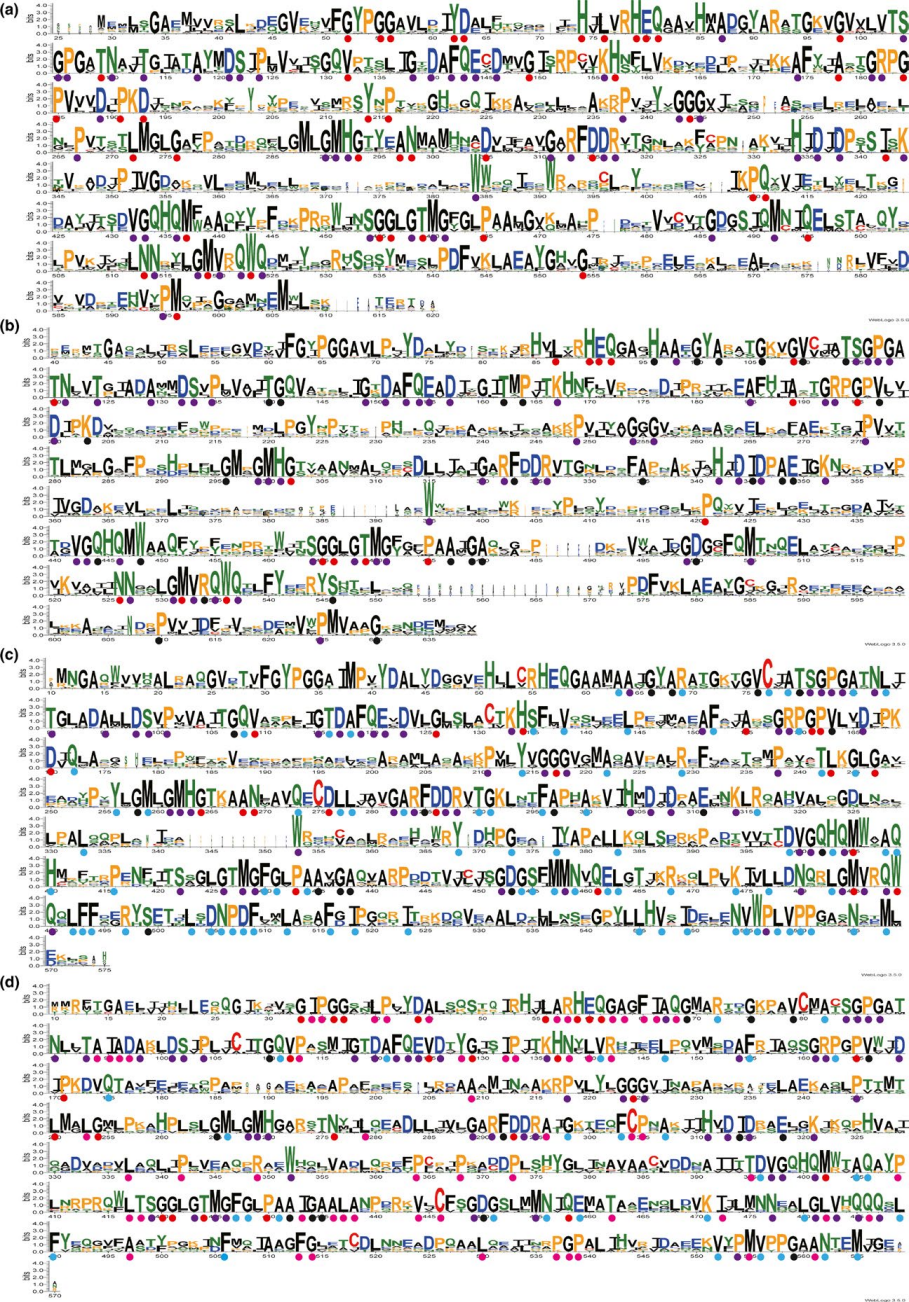
Protein sequence alignments of AHAS III‐A‐L (a), AHAS III‐B‐L (b), AHAS II‐L (c) and AHAS I‐L (d), respectively. The purple solid circles stand for the conserved residues in AHAS‐L; and the red, black, cyan, and carmine solid circles stand for the conserved residues in AHAS III‐A‐L, AHAS III‐B‐L, AHAS II‐L and AHAS I‐L, respectively. AHAS, acetohydroxyacid synthase

Acetohydroxybutyrate synthase genes have been identified and sequenced in a variety of bacterial species. In order to clarify the similarity and difference between different AHASs, the amino acid sequences for a selection of 91 AHAS from the above 70 species were aligned, and the sequence logos were generated in Figure 4. The calculated similarity score of all 91 AHAS‐L is 47.82%, showing 53 identities. While the similarity score of all 91 AHAS‐S is 34.42%, showing 1 identities and 30 conservative substitutions. Those identities were emphasized by purple solid circles in Figure 4. The alignment similarity of AHAS‐S is less than that of AHAS‐L, it may cause by different length of AHAS‐S. AHAS III‐A‐S and III‐B‐S have two ACT‐like subdomains, but AHAS II‐S and AHAS I‐S just have one ACT‐like subdomain. After the C‐terminal ACT‐like subdomain of AHAS III‐A‐S and III‐B‐S were shortened, the calculated similarity score of AHAS‐S was arise to 47.49%. Sequence analysis of the three AHAS‐L in *E. coli* demonstrated that AHAS II‐L and AHAS I‐L have the highest homology (47%), whereas AHAS I‐L and AHAS III‐A‐L have the least homology (39%), and the identity of AHAS II‐L and AHAS III‐A‐L is 44%.

The sequence similarity of proteins in clusters AHAS III‐A‐L, AHAS III‐B‐L, AHAS II‐L and AHAS I‐L are 61.29%, 53.95%, 72.05% and 75.24%, respectively. AHAS are belong to thiamin diphosphate (ThDP)‐dependent family. The common feature of all ThDP‐dependent enzyme is the binding of TPP at the interface of a conserved pyrophosphate and pyrimidine domain (Costelloe et al., 2008). The conserved motif of ThDP‐binding sites, such as G56, E80, T103, P106, Q143, H435, D487, N514, and M519 in AHAS III‐A‐L (Figure 4a) (Bar‐Ilan et al., 2001; Pang, Duggleby, & Guddat, 2002; Baig et al., 2014), were also conserved in AHAS‐L. AHAS‐L contain three domains: α‐, β‐, γ‐ domains. All the conserved ThDP‐binding sites are mainly located on the α‐ and γ‐domains. AHAS are also FAD‐dependent enzyme. AHAS required FAD to maintain the geometry of actives sites in the β‐domain. Although FAD was located in a crevice bounded by all three domains from the same monomer, FAD was associated most closely with the β‐domain (Pang et al., 2002). In the β‐domain of AHAS‐L, five conserved amino acids (R182, L272, H292, R313 and R317 shown in Figure 4a) are important for binding FAD (Pang et al., 2002; Pue & Guddat, 2014). D349 of yeast AHAS has been reported as an FAD‐binding site (Pang et al., 2002), it is conserved in AHAS III‐A‐L (Figure 4a), but not conserved in other types of AHAS‐L (Figure 4b–d); T249 in AHAS I‐L (Figure 4d), corresponding to T271 in Figure 4a, T280 in Figure 4b and T242 in Figure 4c, has also been reported as an FAD binging site (Mitra & Sarma 2008). In the catalytic reaction of all AHAS in Figure 4 a–d, the first binding site is specific for pyruvate. F119 and V486 in AHAS II‐L (Figure 4c) were reported to conjoint mediate substrate binding and specificity (Steinmetz et al., 2010), and these phenylalanine and valine residues were recognized to belong the first substituent pocket in AHAS. From Figure 4, these phenylalanine and valine residues were proved to be both conserved. This phenylalanine residue could be observed in conserved motif “G‐D‐FQE‐D”, meanwhile valine residue was in “G‐V‐Q‐Q” in all four parts of Figure 4. Moreover, AHAS could bind either 2‐ketobutyrate or pyruvate as the second substrate. AHAS II, AHAS III‐A and AHAS III‐B prefer to bind 2‐ketobutyrate rather than pyruvate, while AHAS I has no preference to these two substrates. Residue tryptophan is a key binding site of 2‐ketobutyrate, it is conserved, such as W523 in Figure 4a, W536 in Figure 4b, and W489 in Figure 4c, but this residue is replaced by glutamine in Figure 4d. This glutamine residue (Q486) in Figure 4d kept congruence of the alternative of the second substrate in AHAS. Meanwhile, the loss of high preference for 2‐ketobutyrate was found when *E. coli* AHAS II W489 is changed to leucine. This tryptophan residues was reported as a consequence of similar forward rate constants of carboligation and product release for the alternative second substrates (Tittmann et al., 2005). The conserved arginine residue could be found in the site 317 in Figure 4a, site 336 in Figure 4b, site 288 in Figure 4c and site 295 in Figure 4d. This arginine residue of yeast AHAS was reported to affect the carboligation reaction by increasing electrophilic character of pyruvate. Site‐directed mutations of this arginine residue in *E. coli* AHAS II failed in accepting the second substrate (Engel et al., 2004). In addition, this residue mutations in *Mycobacterium tuberculosis* AHAS all led to a complete loss of AHAS activity (Jung, Cho, Koo, & Yoon, 2015). The conserved sites shown in Figure 4 are important for the catalytic activity. Tuberculosis caused by *M. tuberculosis* is the second leading cause of death, AHAS is recognized as a safe target for developing antimicrobial compounds. Oxygen atoms of benzoyl esters inhibitor form bonds with amino acids K203 and R326 of *M. tuberculosis* AHAS (Wang et al., 2013). These two residues leading to a strong binding affinity between inhibitor and AHAS are both conserved (Figure 4b). In addition, A152 and Q154 of *M. tuberculosis* AHAS are key sites for herbicide binding via hydrophobic interactions. This glutamine is conserved while the alanine is not (Figure 4b), suggesting that different AHAS may be sensitive to different herbicides. Thus, conserved residues shown in Figure 4 provide important information not only for the catalytic activity but also for inhibitor binding of AHAS.

**Figure 5:**
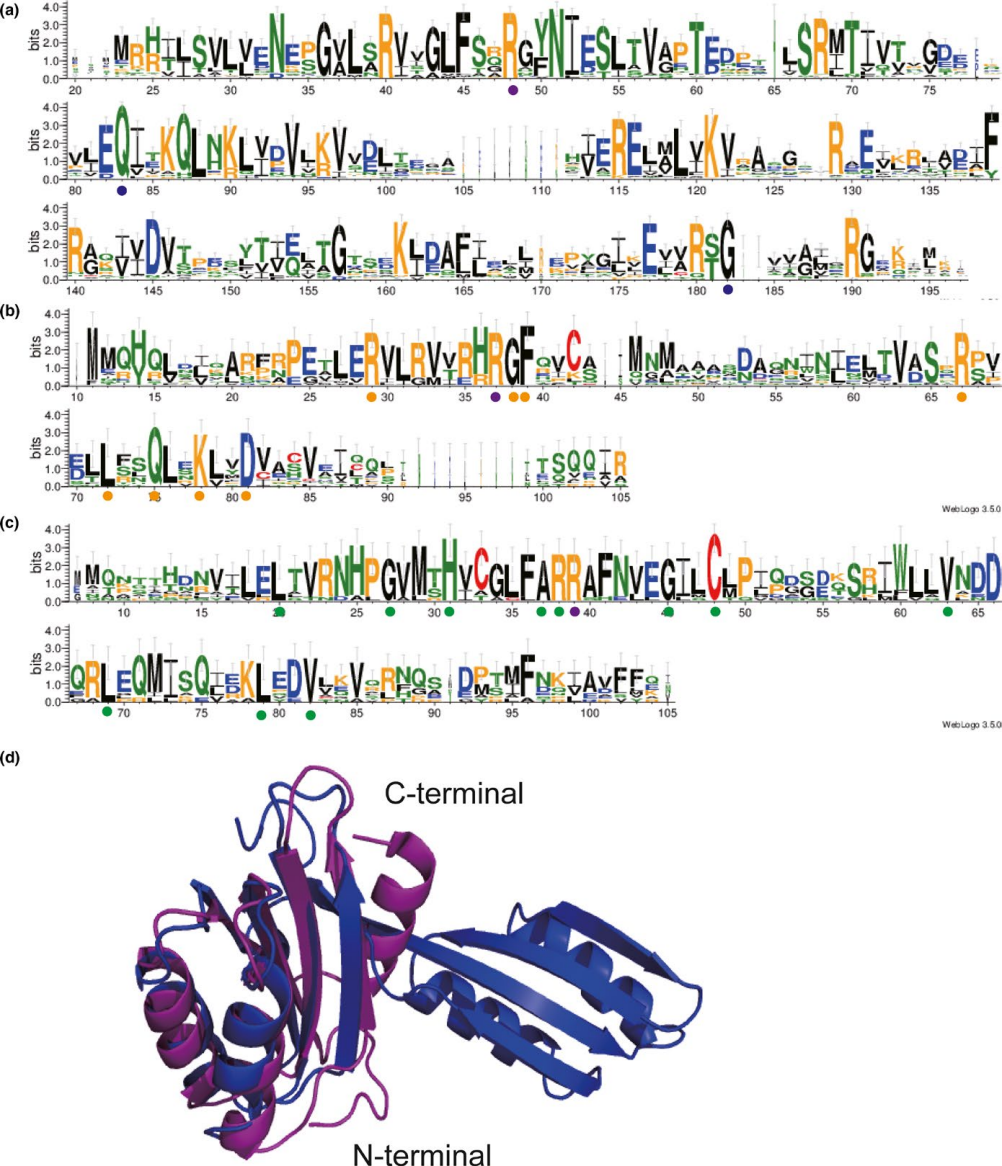
Protein sequence alignments of acetohydroxyacid synthase (AHAS) III‐S (a), AHAS II‐S (b) and AHAS I‐S (c), respectively. The purple solid circles stand for the conserved residues in AHAS‐S, and blue, orange, and green solid circles stand for conserved residues in AHAS III‐S, AHAS II‐S and AHAS I‐S, respectively. (d) Structure alignment of AHAS III‐A‐S (2F1F) and AHAS I‐S (2LVW) from *Escherichia coli* [8, 36]. AHAS, acetohydroxyacid synthase

Except of the large subunit, each AHAS contains a small subunit. Separating AHAS‐S into three parts (Figure 5), the alignment results of AHAS III‐A,B‐S, AHAS II‐S and AHAS I‐S were 44.17%, 49.12% and 61.86%. The conservative substitutions of each AHAS‐Ss were shown by blue, orange and green solid circle. AHAS‐S contain one or two ACT‐like subdomains in metabolic enzymes. ACT family domains were always combined with other domains to provide easily regulated enzymes. The structure of AHAS‐S had been crystallized in the species of *E. coli* (Kaplun et al., 2006; Karanth & Sarma, 2012), *Thermotoga maritima* and *Nitrosomonas europea* (Petkowski et al., 2007). The alignment of AHAS III‐A‐S and AHAS I‐S both from *E. coli* were showed in Figure 5. Residues from AHAS III‐A ‐S are shown in blue and residues from AHAS I‐S are shown in purple. It depicted that AHAS III‐A‐S and AHAS I‐S have the similar three dimensional structure. The protein of AHAS‐S in species was a dimer, with two βαββαβ ferredoxin topology in each monomer. The effector binding sites could be located tentatively in the interface between the dimer of the first ACT‐like subdomains. This dimer were remarkably close to the C‐terminal of 3‐phosphoglycerate dehydrogenase. The highly conserved structures of AHAS III‐A‐S and AHAS I‐S suggested that they should be evolved from the same ancestor. Mostly conserved glycine residue could be found in site G36 in Figure 5a and G27 in Figure 5c, but it was not conserved in site 56 in Figure 5b. When changing conserved G36 of *E. coli* AHAS III, AHAS III became valine‐insensitive (Kaplun et al., 2006). These found are consistent with that AHAS II was not sensitive to feedback inhibition, while AHAS I and AHAS III could be inhibited by valine.

### 3.4 Chromosomal organization of genes relevant to the genes encoding AHAS in bacteria

**Figure 6:**
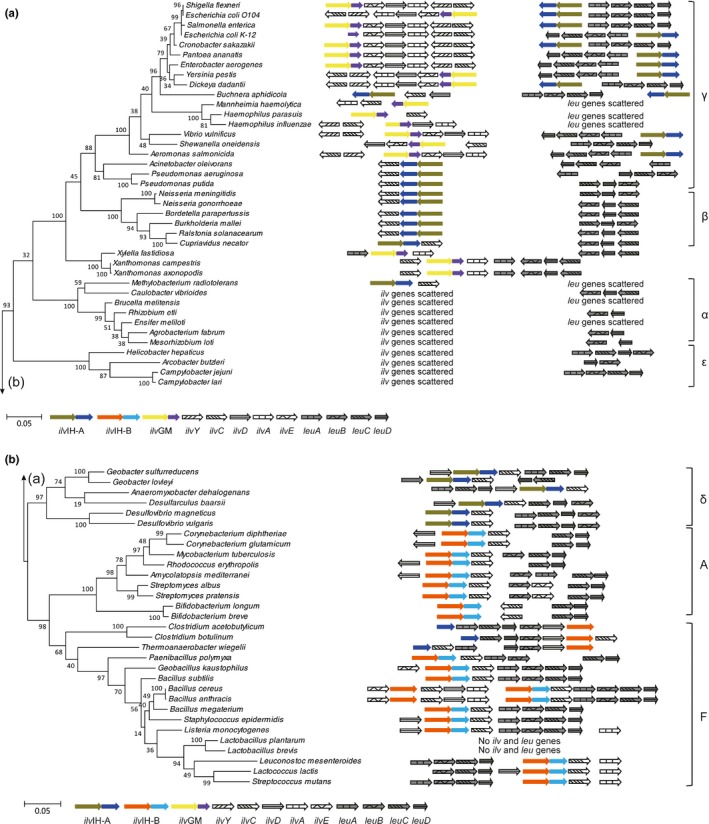
Gene organization of branched chain amino acids genes. Structure and organization of isoleucine, valine, and leucine biosynthetic genes of the 70 bacteria correlated with their phylogenetic position. (a) stands for species in α‐, β‐, γ‐, ε‐proteobacteria. (b) stands for species in δ‐ proteobacteria, firmicutes and actinobacteria

The parallel pathways of BCAA biosynthesis are catalyzed by three common enzymes. The first of them is acetohydroxyacid synthase (AHAS). Apart to AHAS, valine and isoleucine is form with the conversion of either acetolactate or 2‐aceto‐2‐hydroxybutyrate catalyzed by ketol‐acid reductoisomerase (KARI, *ilv*C), and reactions by dihydroxyacid dehydratase (DHAD, *ilv*D) and transaminase (TA, *ilv*E) [4, 5]. In addition, four enzymes coding by *leu*A, *leu*B, *leu*C and *leu*D channel 2‐ketoisovalerate toward leucine biosynthesis. The appearance of multi‐copied genes is often parallel to their presence within operons, the analysis of the aspartokinases in γ‐proteobacteria have proved it (Fondi et al., 2007b). This raises the question whether the structure and distribution of duplicated copies of AHAS genes might somehow be correlated with their organization in the bacterial genome. Therefore, we analyzed the organization of all the genes of the branched chain amino acid biosynthesis in all the 70 species. The organization of *ilv* genes in bacteria partly focus on four genes: *ilv*A, *ilv*C, *ilv*D, and *ilv*E. The *leu* biosynthetic genes contain *leu*A, *leu*B, *leu*C, and *leu*D. The obtained data were shown in Figure 6.

At the bottom of Figure 6a, except *Methylobacterium* strains that own a *ilv*IH‐A*‐ilvC* operon, AHAS III‐A and the genes encoding other four enzymes involved in isoleucine and valine biosynthesis (*ilvA*,* ilvC*,* ilvD* and *ilvE*) were scattered on the chromosome of bacteria belonging to α‐ and ε‐ subdivisions of proteobacteria. Moreover, in *Brucella*,* Methylobacterium* and *Mannhiemia* all the *leu* biosynthetic genes were split. In β‐ and δ‐proteobacteria branches, *ilv*IH‐A was clustered with *ilvC* to form a bicistronic *ilv*IH‐A*‐ilvC* operon. The *leu* gene order was *leu*C‐*leu*D‐*leu*B as usual. These two operon were distal from each other. However, the scenario was completely different in the branches of γ‐proteobacterial at the top of Figure 6a. For example, *E. coli* and *Cronobacter sakazakii* maintained a consensus operon gene orders *leu*ABCD together with *ilv*IH‐A. In Figure 6b, *ilv*IH‐B and *ilv*C were remain together in firmicutes and actinobacteria branches. Whereas, in firmicutes group, dramatic scrambling of gene order was apparent, *leu* pathway genes *leu*ABCD were observed to follow the AHAS III‐A‐*ilv*C operon. In some cases, *leu* pathway genes had been inserted into AHAS III‐B‐L and AHAS III‐B‐S. Thus, in *Clostrisium* and *Theroanaerobacter*,* ilv*I‐B and *ilv*H‐B were split aside from the genome. AHAS II was endowed within a 4‐cystronic operon, in the same relative gene order (*ilv*GM‐ilv*EDA*). No related genes were observed accompanying with AHAS III‐A. At first, single AHAS was more possible to situate in the genome. With the increase demand of branched chain amino aicds, AHAS and *ilv*C were clustered together. Then like the γ‐proteobacteria and firmicute branches showed, the *leuABCD* operon was located nearby AHASs. Different from firmicute AHAS III‐B, γ‐proteobacteria species are organized as two divergent transcribed groups, *ilv*IH‐A‐*leu*ABCD and *ilv*GM‐*ilv*EDA. Meanwhile, in Figure 6b, the duplicated AHAS III‐B of *Bacillus cereus* in firmicute were inserted into two operons combined by common genes *ilv*E and *ilv*C, and different genes *ilv*D, *ilv*A and *leu*ABCD, respectively. Horizontal gene transfer was deduced to occur between γ‐proteobacteria and firmicute. If so, the occurrence of AHAS II might be more specificity to substrate than AHAS III‐B. AHAS II bind 2‐ketoacid twofold higher than AHAS III‐B supported this hypothesis (Bar‐Ilan et al., 2001). Sequence alignment of *ilv*C in bacteria showed that *ilv*C in γ‐proteobacteria was differ from the genes in other classes. Thus, except for horizontal gene transfer and duplication, the re‐construction of AHAS operon should have been occurred during evolution of γ‐proteobacteria species.

## 4 DISCUSSION

AHAS belongs to the pyruvate oxidase‐like subfamily of ThDP‐dependent family, which catalyze the decarboxylation of α‐ketoacids (Chang & Cronan, 1988). In pyruvate oxidase‐like subfamily, the enzymes that have been crystallized share similar three‐dimensional structure. Evolutionary pathway of ThDP‐dependent family enzymes suggests that the α‐ and γ‐domain of pyruvate oxidase‐like subfamily are arisen from a duplication event of a common ancestor. The similar position of NADP^+^ in dIII of proton‐translocating transhydrogenase (Mather, Singh, van Boxel, White, & Jackson, 2004) and FAD in pyruvate oxidase‐like subfamily confirms a hypothesis that the β‐domain is derived from dIII and its ancestor (Duggleby, 2006). Then the β‐domain is supported to be recruited during the domain arrangement in the evolutionary history. Pyruvate decarboxylase, indolepyruvate decarboxylase and benzoylformate decarboxylase are FAD‐independent enzymes among pyruvate oxidase‐like subfamily, their β‐domains have already lost the property of binding nucleotides (Bornemann, 2002; Pang et al., 2004; Costelloe et al., 2008). From phylogenetic tree constructed according to the sequences of pyruvate oxidase‐like subfamily, FAD‐independent pyruvate decarboxylase is not in the same clade with AHAS, while pyruvate oxidase and AHAS are in the same cluster (Liu, Li, & Wang, 2016). Crystal structures of AHAS‐L from yeast and *Arabidopsis thaliana* have been reported (Pang et al., 2002; McCourt, Pang, King‐Scott, Guddat, & Duggleby,^2006^), but no crystal structure of AHAS‐L from bacteria is available yet. According to similar sequence, structure, common cofactors, same substrate and substitutable function, bacterial AHAS is likely to be evolved from a common ancestor with pyruvate oxidase which FAD participate in catalytic cycles (Chang & Cronan, 1988; Muller & Schulz, 1993).

**Figure 7:**
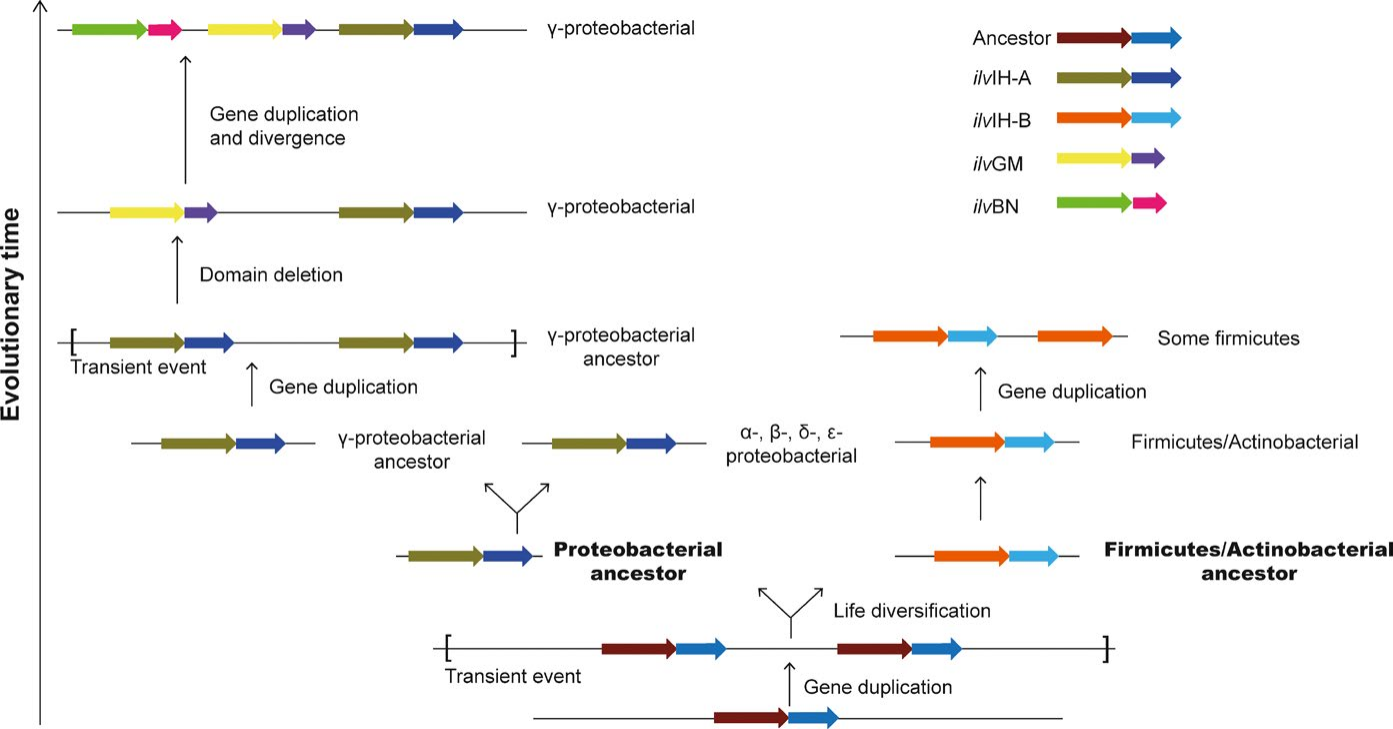
The evolution model and diversification of acetohydroxyacid synthase in bacteria

On the basis of the sequences, structure and phylogenetic analysis, a plausible evolutionary model for AHAS was proposed. The model in Figure 7 predicted that the ancestor possessed only a single copy of AHAS. In archaea, only a single pair of genes encoding AHAS exist, and the two genes are next to each other in the genome. Since the occurrence of AHAS was thought before species generation. Followed with new species generation, the ancestor of gene AHAS divergent into AHAS III‐A and AHAS III‐B with species evolutionary. A duplication of AHAS may have taken place to generate AHAS I‐II, the superfluous C‐terminal of AHAS I‐II‐S in this new copy was deleted. With the divergence of new species, one or two of the genes encoding for AHAS and AHAS I‐II were deleted from the genome. The similar deletion events along with new species generation were common, these were also found in other enzymes, such as threonine dehydratase in isoleucine biosynthesis pathway and UDP‐2,3‐diacylglucosamine pyrophosphatase in Kdo_2_ lipid A biosynthesis pathway (Opiyo, Pardy, Moriyama, & Moriyama, 2010; Yu et al., 2013). Finally, among the species of γ‐proteobacteria, the gene AHAS II might be duplicated, generating AHAS I. It could be supported by the common special character that AHAS II and AHAS I could form chiral arylacyl carbinols as precursors for pharmaceutical syntheses (Chipman et al., 2009). The finding that AHAS I only showed in γ‐proteobacteria, strongly suggested that this duplication event might have occurred in a relatively short time. The expection of gene AHAS I which were also observed in *Desulfovibrio* of δ‐proteobacteria and one species of firmicute, suggesting that the horizontal gene transfer could also occur in the evolution of AHAS.

This evolutionary model of AHAS is consistent with the published theories, which suggested that new enzymes are usually evolved from enzymes with similar biochemical function rather than in the same biosynthetic pathway (Alves, Chaleil, & Sternberg, 2002; Chatterjee & Yuan, 2006). Meanwhile, the biological significance of this duplication and deletion events might rely on the “patchwork” hypothesis on the origin and evolution of metabolic pathways (Fani & Fondi, 2009). According to this hypothesis, the ancestral cells were almost heterotrophic organisms which just have to do a minimum biosynthesis in the primordial soup where contains different nutrient compounds (Miller, 1953). The primitive cells contain small genomes which were probably composed by about 1,000–1,500 genes (Ouzounis, Kunin, Darzentas, & Goldovsky, 2006). Therefore, AHAS were more possible to start from a single gene. After paralogous gene duplication and divergence, thousands of different enzymes with narrow specificity were allowed to form well defined routes which could form distinct molecules (Fondi et al., 2007b; Fani & Fondi, 2009). It was equal to the increased copies of AHAS. And AHAS II located with the isoleucine biosynthesis operon in the *E. coli* genome showed the highest preference to 2‐ketobutyrate than pyruvate as the second substrate (Barak, Chipman, & Gollop, 1987). To better adapting environment lacking appropriate carbon source, AHAS I in *E. coli* was reported to be most useful where pyruvate is inadequate (Dailey & Cronan, 1986). Exponentially growing *E. coli* cells contain about 300 μmol/L pyruvate and 3 μmol/L 2‐ketobutyrate, in which AHAS I would produce almost entirely acetolactate, however, AHAS II and AHAS III‐A would synthesize acetolactate and acetohydroxybutyrate in the ratio required for protein synthesis (Barak et al., 1987). The first ACT‐like subdomain of AHAS III‐A was shown to be the minimum activation motif and it regulated all AHAS (Zhao, Niu, Wen, & Xi, 2013). Since the second ACT‐like subdomain of AHAS II, B‐S was more likely to be deleted by AHAS III‐A‐S. The phylogenetic distribution in Figure 2 strongly suggests that the duplication of AHAS coding genes can be traced within γ‐proteobacteria or soon after the divergence of the γ‐proteobacteria ancestor from α‐, β‐, δ‐, and ε‐proteobacterial. This hypothesis is also confirmed by the sequences alignment of *ilv*C. The γ‐proteobacteria branch is separate from the other three clusters.

The ancestral copy might have been under the control of multiple different regulatory signals. Once a “new” gene inserts the genome and becomes part of a pre‐existing metabolic pathway, it is plausible to become coregulated with the other genes belonging to the same metabolic pathway (Fani, Brilli, & Liò, 2005). In some cases, coregulation of genes of the same biosynthetic route is achieved by organizing genes in operon structures, even though coregulation may also be obtained by the regulon (Fondi et al., 2007b). In general, if the model proposed and its biological significance are correct, AHAS would become increasingly more sensitive to specific regulatory signals during the successive evolutionary divergence. It seems plausible that expression of AHAS II‐ilvEDA operon of *E. coli* is controlled by the global regulator leucine responsive protein. While the single AHAS III‐B in *C. glutamicum* is out of the control (Park & Lee, 2010). Data obtained in Figure 6 suggest that the production of genes coding for enzymes specific of a single metabolic pathway coincides with their presence within a polycistronic transcriptional unit that includes all (or at least some of) the other genes of that route. Concerning the timing of the operons construction, the comparative analysis revealed that the “gene duplication” occurring in γ‐proteobacteria appears to be coincident with gene clustering and the formation of operons of different length.

## CONFLICT OF INTEREST

The authors have no conflict of interest to declare.
