# Supplementary material for: Molecular evolution of acetohydroxyacid synthase in bacteria
Source: Microbiologyopen. 2017 Aug 6;6(6):e00524. doi: 10.1002/mbo3.524 (PMC5727371; doi:10.1002/mbo3.524)

**Molecular evolution of** **acetohydroxyacid synthase in bacteria**

Yadi Liu^1,2^, Yanyan Li^1^ and Xiaoyuan Wang^1,2,3^

^1^State Key Laboratory of Food Science and Technology, ^2^School of Biotechnology, and ^3^Synergetic Innovation Center of Food Safety and Nutrition, Jiangnan University, Wuxi 214122, China.

**Running Head: Acetohydroxyacid synthase in bacteria**

Corresponding author：

Prof. Xiaoyuan Wang

State Key Laboratory of Food Science and Technology

Jiangnan University

1800 Lihu Avenue

Wuxi 214000

China

Tel: +86-510-85329236

Fax: +86 510 85329236

E-mail: xwang@jiangnan.edu.cn

**Table S1.** The list of the representative 70 species that contain AHAS used in this study.

| NO | Species | KEGG | 16srDNA |
| --- | --- | --- | --- |
| **γ-proteobacteria** | | | |
| 1 | *Escherichia coli str. K-12* | eco | S000529092 |
| 2 | *Escherichia coli O104* | esl | S004062442 |
| 3 | *Dickeya dadantii* | ddd | S002236689 |
| 4 | *Salmonella enterica* | stm | S000438765 |
| 5 | *Yersinia pestis* | ype | S000539393 |
| 6 | *Shigella flexneri* | sfl | S000497725 |
| 7 | *Buchnera aphidicola* | buc | S002287201 |
| 8 | *Enterobacter aerogenes* | eae | S004067104 |
| 9 | *Cronobacter sakazakii* | esa | S002289650 |
| 10 | *Pantoea ananatis* | pam | S000021356 |
| 11 | *Mannheimia haemolytica* | mhx | S004061944 |
| 12 | *Xylella fastidiosa* | xfa | S000497684 |
| 13 | *Xanthomonas campestris* | xcc | S000539136 |
| 14 | *Xanthomonas axonopodis* | xao | S003710875 |
| 15 | *Vibrio vulnificus* | vvy | S002287482 |
| 16 | *Shewanella oneidensis* | son | S000392860 |
| 17 | *Acinetobacter oleivorans* | acd | S002287650 |
| 18 | *Pseudomonas aeruginosa* | pae | S000386395 |
| 19 | *Pseudomonas putida* | ppu | S002287386 |
| 20 | *Aeromonas salmonicida* | asa | S002288221 |
| 21 | *Haemophilus influenzae* | hin | S002289475 |
| 22 | *Haemophilus parasuis* | hap | S002287216 |
| **β-proteobacteria** | | | |
| 23 | *Neisseria meningitidis* | nme | S002287455 |
| 24 | *Neisseria gonorrhoeae* | ngo | S004063217 |
| 25 | *Ralstonia solanacearum* | rso | S000540283 |
| 26 | *Burkholderia mallei* | bma | S002287602 |
| 27 | *Bordetella parapertussis* | bpa | S000528611 |
| 28 | *Cupriavidus necator* | cnc | S002287849 |
| **ε-proteobacteria** | | | |
| 29 | *Helicobacter hepaticus* | hhe | S004068723 |
| 30 | *Campylobacter jejuni* | cje | S000721402 |
| 31 | *Campylobacter lari* | cla | S002287278 |
| 32 | *Arcobacter butzleri* | abu | S002288142 |
| **δ-proteobacteria** | | | |
| 33 | *Geobacter sulfurreducens* | gsu | S000437158 |
| 34 | *Geobacter lovleyi* | glo | S000490893 |
| 35 | *Desulfovibrio vulgaris* | dvu | S002290353 |
| 36 | *Desulfovibrio magneticus* | dma | S000413803 |
| 37 | *Anaeromyxobacter dehalogenans* | ade | S000392793 |
| 38 | *Desulfarculus baarsii* | dbr | S002289416 |
| **α-proteobacteria** | | | |
| 39 | *Mesorhizobium loti* | mlo | S004062516 |
| 40 | *Brucella abortus* | bmf | S000617642 |
| 41 | *Methylobacterium radiotolerans* | mrd | S002289014 |
| 42 | *Caulobacter crescentus CB15* | ccr | S000497740 |
| 43 | *Agrobacterium fabrum* | atu | S002288566 |
| 44 | *Rhizobium etli CFN 42* | ret | S000644968 |
| 45 | *Sinorhizobium meliloti* | sme | S000099383 |
| **Firmicute** | | | |
| 46 | *Bacillus subtilis* | bsu | S000529480 |
| 47 | *Bacillus cereus* | bca | S002287168 |
| 48 | *Bacillus anthracis* | ban | S002287776 |
| 49 | *Bacillus megaterium* | bmq | S002290379 |
| 50 | *Staphylococcus epidermidis* | sep | S004063253 |
| 51 | *Listeria monocytogenes* | lmo | S000540245 |
| 52 | *Streptococcus mutans* | smu | S002290690 |
| 53 | *Paenibacillus polymyxa* | ppm | S004068843 |
| 54 | *Leuconostoc mesenteroides* | lme | S002288561 |
| 55 | *Lactococcus lactis* | lla | S002290990 |
| 56 | *Lactobacillus*brevis | lbr | S002288203 |
| 57 | *Lactobacillus plantarum* | lpl | S000540305 |
| 58 | *Geobacillus kaustophilus* | gka | S000528507 |
| 59 | *Clostridium botulinum* | cbk | S003917501 |
| 60 | *Clostridium acetobutylicum* | cac | S002287075 |
| 61 | *Thermoanaerobacter wiegelii* | twi | S004062571 |
| **Actinobacteria** | | | |
| 62 | *Mycobacterium tuberculosis* | mtu | S000528623 |
| 63 | *Corynebacterium glutamicum* | cgl | S004066010 |
| 64 | *Corynebacterium diphtheriae* | cdi | S002287989 |
| 65 | *Streptomyces albus J1074* | salb | S004063576 |
| 66 | *Bifidobacterium longum* | blo | S002287617 |
| 67 | *Bifidobacterium breve* | bbru | S004066430 |
| 68 | *Rhodococcus erythropolis* | rer | S002289921 |
| 69 | *Amycolatopsis mediterranei* | amd | S002288880 |
| 70 | *Streptomyces pratensis* | sfa | S004066449 |

**Figure S1**


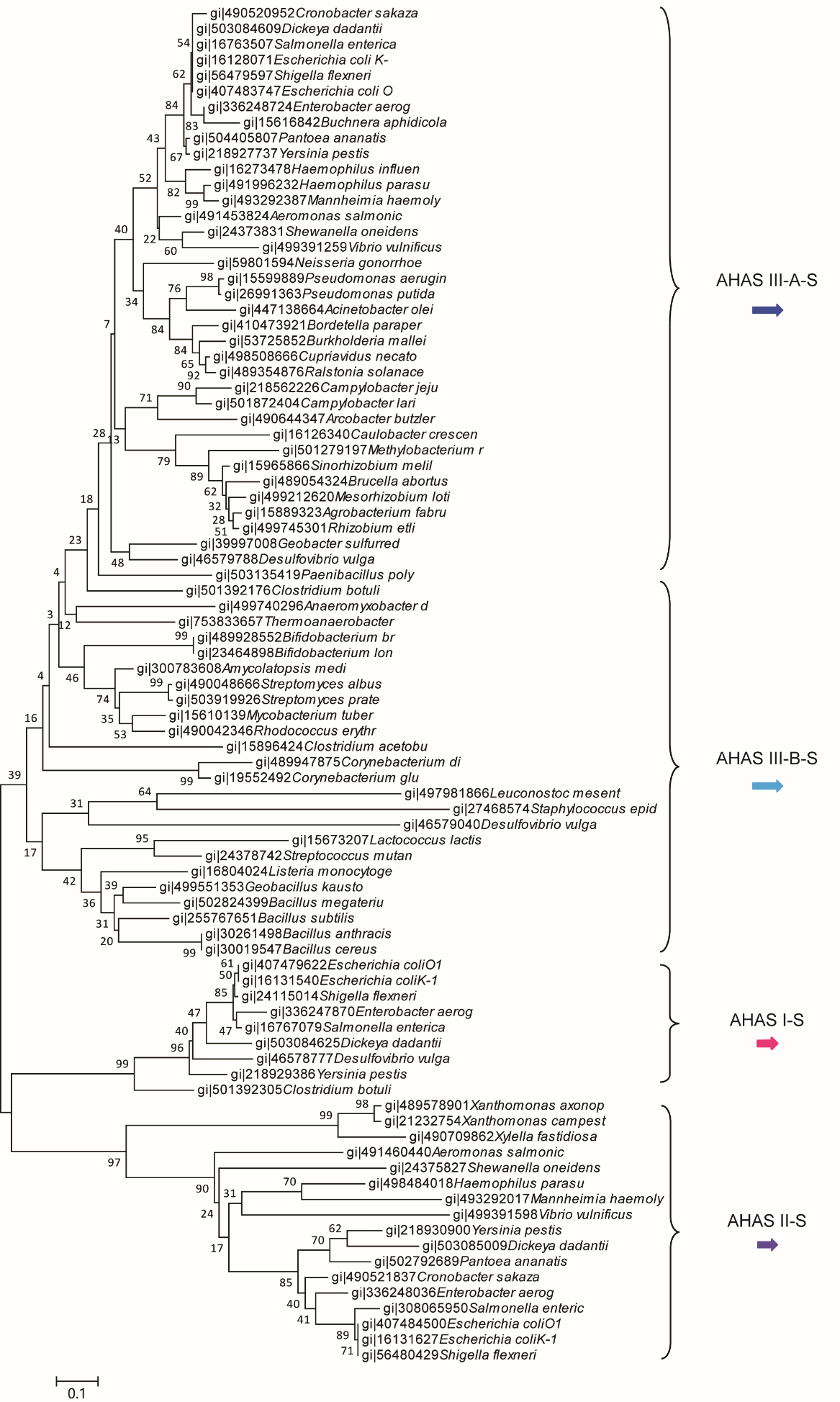

Supplement: Supplementary file 1 [file MBO3-6-na-s001.docx]
